# Use of Complementary Natural Feed for Gastrointestinal Nematodes Control in Sheep: Effectiveness and Benefits for Animals

**DOI:** 10.3390/ani9121037

**Published:** 2019-11-27

**Authors:** Fabio Castagna, Ernesto Palma, Giuseppe Cringoli, Antonio Bosco, Nancy Nisticò, Giada Caligiuri, Domenico Britti, Vincenzo Musella

**Affiliations:** 1 Department of Health Sciences, University of Catanzaro “Magna Græcia”, CISVetSUA, 88100 Catanzaro, Italy; palma@unicz.it (E.P.); nancynistico@libero.it (N.N.); jada.caligiuri@gmail.com (G.C.); britti@unicz.it (D.B.); musella@unicz.it (V.M.); 2 Department of Veterinary Medicine and Animal Productions, University of Naples Federico II, 80137 Napoli, Italy; cringoli@unina.it (G.C.); boscoant@tiscali.it (A.B.)

**Keywords:** gastrointestinal nematodes, sheep, FLOTAC technique, natural vegetable mixture, phytotherapy, ivermectin, anthelminthic effectiveness

## Abstract

**Simple Summary:**

The massive and frequent use of drugs to control helminthiasis in sheep might lead to anthelmintic resistance and the presence of residues in the environment. A solution must be found to reduce these risks and natural extracts could be a valid alternative to drugs. The authors report the results of an in vivo study on the effectiveness of a complementary feed, based on natural extracts, without residual risk for the environment, registered for gastrointestinal nematodes control in sheep and comparing its efficacy to the drug ivermectin. This study shows the better efficacy of ivermectin as compared with natural formulations and highlights the importance of in vivo studies for the evaluation of the natural mixtures registered for the treatment of gastrointestinal nematode infections

**Abstract:**

The treatments of gastrointestinal nematodes (GIN) infection in sheep is almost exclusively based on the use of synthetic drugs. In some European regions the intensive use of antiparasitic drugs is leading to widespread development of anthelmintic resistance (AR). Currently in southern Italy AR is rare, but a constant monitoring of anthelmintic efficacy and the use of effective alternative therapies is strongly recommended. The aim of our study was to evaluate the effectiveness of a complementary natural feed (natural vegetable mixture), based on natural extracts, registered for GIN treatment in sheep, and its comparison with the drug ivermectin. The study was conducted in two sheep breeding farms in southern Italy and 75 sheep were divided in groups of 15 animals each (treated and untreated groups), homogeneous by GIN eggs per gram (EPG) of faeces, using the natural anthelmintic administered at full dose (10 g/sheep/orally) in the first breeding and at double dose (20 g/sheep/orally) in the second. In the latter we compared the effectiveness of mixture with ivermectin administered at full dose (200 μg/kg/BW). To determine the effectiveness, individual faecal samples were collected to evaluate the faecal eggs count (FEC) using FLOTAC technique and FEC reduction (FECR) on different days. The formula used FECR = 100 × (1 − (T2/C2)), based on the comparison of post-treatment EPG mean of the treated and untreated group (T2 and C2, respectively), is the one recommended by World Association for the Advancement of Veterinary Parasitology (W.A.A.V.P.) guidelines to monitor drug efficacy against GIN in livestock. The results reported that complementary natural feed, at two different dosages, was ineffective against GIN, while the drug, at conventional dosage, showed good anthelmintic efficacy, also confirming the importance of in vivo effectiveness studies.

## 1. Introduction

Gastrointestinal nematode (GIN) infections are a common constraint in pasture-based herds and can cause a decrease in animal health, productivity and farm profitability [1]. The impact of GIN infection in sheep is linked to clinical signs associated with the infection and also to subclinical economic losses due to decreased growth and milk production [2,3]. Nowadays these infections remain a major constraint to ruminants’ health, welfare and productive performance worldwide [4,5].

Gastrointestinal nematode control programs are mainly based on a combination of both animal management practices, pastures management and the use of anthelmintic drugs [6]. The intensive use of synthetic anthelmintics for treating and control of GIN in the sheep farms has led to the widespread development of resistance to one or more anthelmintic drug classes at the same time [7]. Anthelmintic resistance (AR) in human and animal pathogenic helminths has been spreading in prevalence and severity to a point where multidrug resistance (MDR) against the three major classes of anthelmintics—the benzimidazoles, imidazothiazoles and macrocyclic lactones—has become a global phenomenon in gastrointestinal nematodes of farm animals [8].

The development of resistance to all the older anthelmintic groups in sheep is a serious increasing problem [9,10] and resistant GIN are a great hazard for sheep flocks and farmers, at the point that, sometimes, the inability to control the parasites infestation results in the farm closures or culling of entire flocks [9,11]. Anthelmintic resistance and multiple drug resistance is very problematic in Australia, New Zealand, South Africa and many Latin American countries [12,13].

This phenomenon is recently becoming a threat also in Europe [14]. The current situation in Europe indicates that ivermectin and moxidectin are the most effective drugs, while benzimidazoles, followed by levamisole, have a markedly reduced efficacy against GIN affecting sheep. While single AR appears more frequent than MDR in individual sites, the finding of parasite populations that may survive to the treatment with more than one anthelmintic class is of high concern. Despite total anthelmintic failure already reported in Scotland, these still represent isolated cases in Europe [14]. Resistance or suspected resistance was recorded on all farms from France where benzimidazoles have been tested, in Greece and Italy, AR against levamisole and benzimidazoles was observed on some farms, with MDR involving both anthelmintics was proved on few sites of these countries [7,14]. Anthelmintic resistance is rare in southern Italy and it is likely that correct management practices strongly reduce the insurgence of resistance mechanisms. Thus, a constant monitoring of the efficacy of anthelmintics in sheep in southern Italy is strongly recommended [15].

The frequent and indiscriminate use of anthelmintic drugs, besides favouring the phenomena of drug resistance could have a significant environmental impact, deriving from the presence of drug residues excreted with animal faeces [16]. Some studies highlight the potential risk deriving from the massive use of anthelmintic drugs, which have a long persistence in the environment [17] and that can be harmful towards many invertebrate species; especially those belonging to orders such as Dictyoptera, Anoplura, Homoptera, Thysanoptera, Colaptera, Siphonaptera, Diptera, Lepidoptera and Hymenoptera, as well as some species of fish [18,19].

There is an urgent need to seek alternative or complementary solutions to the control of parasitic nematodes of ruminants [20,21] and because of the widespread resistance to synthetic anthelmintics, there is a strong impetus to explore novel approaches for a more integrated management of these infections [22].

Before the creation of synthetic anthelmintics by chemical companies in the mid-20th century, humans relied on plants to control livestock intestinal parasites [23]. Several printed books dating back to 17th–19th century document the use and a comprehensive descriptions of plants fed to livestock to treat parasitosis [24].

Many studies confirm the effectiveness of some plants traditionally known for their antiparasitic properties in ruminants [25,26] and their use can be considered for combined treatment with synthetic anthelmintics [27]. However, the studies performed using commercial phytotherapeutics are rare. These natural mixtures, often sold as complementary feeds, and not as veterinary drugs, contain extracts of different plants in unspecified proportions [26].

Therefore, the aim of the current study was to evaluate the anthelmintic effectiveness of a registered natural product for GIN control in sheep. Moreover, we compared this commercially available product with a well-established synthetic compound (ivermectin).

## 2. Materials and Methods

### 2.1. Ethical Approval and Study Area

The experimental design as well as all animal treatments were approved by the ethical committee of the University of Catanzaro “Magna Græcia” with the approval number 97 of 09/10/2015.

The study was conducted in the Calabria region of southern Italy, in a territory with a Mediterranean climate. In this region, the sheep farming is very widespread, mostly in marginal areas unsuitable for agricultural production, and represents an important economic resource for the agri-food sector, particularly for local dairy products.

### 2.2. Study Farms and Animals

The study was conducted between February 2016 and April 2017 in two dairy sheep farms. Sheep farms selection was mainly driven by the availability of the farmer.

At 21 days from the tests (D-21) in the sheep farms the breeding system and the animals raised were examined.

The breeding system and the size of the flock were similar. The farms were specialized in dairy production and practiced a breeding system based on keeping animals on the pasture. The mean size of the flocks was 120 sheep of Comisana breed and dairy crossbreeds (e.g., Sarda × local regional breeds).

The animals used for the trials were homogeneous for age (3.5 years ± 0.7), body weight (48 kg ± 1.9) and grazing season, without any anthelmintic treatments in at least six months.

### 2.3. Faecal Sampling and Anthelmintics Administration

The efficacy of a complementary natural feed (natural vegetable mixture) on the animals was evaluated without residual risk for the environmental and zero-day withholding period for milk and meat. The product is commercially available and registered for treatment of GIN infection in sheep. Efficacy of the treatment was also compared with the ivermectin drug-based treatments.

The drug and the natural mixture used in this study were IVOMEC (Merial) and Lilium O. New (Rosa canina c.l.). These products were administered at the doses provided by the manufacturers for the purposes indicated, in full respect of animal welfare.

The natural vegetable mixture in its composition presented: water, extracts and essential oils of herbs belonging to the *Compositae*, *Cesalpinacae*, *Liliacae*, *Bromeliaceae* and *Labiatae* families, capric/caprionic acid, lactic acid, propylene glycol and emulsifier. These plants bring active ingredients with anthelmintic activity, particularly: essential oils, resins, tannins, organic acids and mucilages. The feed analytical constituents were: moisture matter (93.3%), crude ashes (1.35%), crude lipids (0.16%), crude proteins and traces of raw cellulose. The manufacturer recommends the following dosage: 7 g/lamb (from 1 to 10 kg) in single administration, 10 g/sheep in single administration, 20 g/sheep under serious infestations in single administration.

Tests were run on groups of sheep (15 animals per group) using the natural anthelmintic administered at full dose of 10 g/sheep in the first farm and at double dose of 20 g/sheep in the second farm.

In the last breeding the effectiveness of natural anthelmintic was compared with an ivermectin drug administered at conventional dose of 200 µg per kilogram of live weight.

In the first sheep farm (SF1) faecal samples from 60 sheep were taken for laboratory exams (day –7). From this parasitological screening it was possible to select the sheep (30 animals), homogeneous for parasitic intensity, for the test groups (15 for group), as follows: NG10, treated with natural anthelmintic administered 10 g/sheep orally as a single dose; CG1, untreated. The first effectiveness study was made in March 2016 with the following timing. At the day zero (D0) the animals were grouped and faecal samples were collected for faecal egg count (FEC). The anthelmintic treatment was then administered. At day 7 (D7) faeces were sampled for FEC. At day 14 (D14) and day 21 (D21) faeces were collected and the faecal egg count reduction (FECR) calculated for the evaluation of anthelmintic effectiveness.

In the second sheep farm (SF2), faeces from 90 sheep were taken for laboratory exams (D–7). The parasitological screening enabled the grouping of the sheep according to homogeneity for parasitic intensity. Each test group was composed of 15 animals, and the diverse groups were labelled as follows: NG20, treated with natural anthelmintic administered at double dose of 20 g/sheep orally in a single administration; IVG, treated with ivermectin drug administered as a single dose of 200 µg per kilogram of live weight subcutaneously; CG2, untreated.

The second study was made in March 2017 and the timing was: D0: groups formation, sampling faeces for FEC and treatment; D7: sampling of faeces for FEC; D14 and D21 sampling of faeces and calculation of FECR for the evaluation of anthelmintic effectiveness.

### 2.4. Laboratory Examination

Individual faecal egg counts (FEC) were determined using the FLOTAC dual technique [28] with a sensitivity of 2 eggs per gram (EPG) of faeces, using a sodium chloride-based flotation solution (specific gravity = 1.200).

In addition, for each group a pooled faecal culture was performed at D0, following the protocol described by the Ministry of Agriculture, Fisheries and Food (MAFF) [29]. Developed third-stage larvae (L3) were identified using the morphological keys proposed by van Wyk and Mayhew [30]. Identification and percentages of each nematode genera were conducted on 100 L3; if a sample had 100 or less L3 present, all larvae were identified. So, on the total number of larvae identified, it was possible to give the percentage of each genus.

### 2.5. Anthelmintic Efficacy

The arithmetic mean EPG was calculated as recommended by the WAAVP guidelines for evaluating the efficacy of anthelmintic in ruminants. The treatment group, instead, was described by percent efficacy (%) calculated in terms of FECR on the different sampling days [31].

The formula used to evaluate the anthelmintic efficacy (based on the comparison of the post-treatment EPG mean) was FECR = 100 × (1 − (T2/C2)), where T2 represents the mean post-treatment FEC of the treated group, and C2 represents the mean post-treatment FEC of the untreated control group [31]. The arithmetic mean was used and 95% confidence intervals (CI) were calculated by using variance in treatment and control groups as set out in Coles et al. [31].

## 3. Results

### 3.1. Coprocultures

In SF1, the following GIN genera were detected at D0 (pre-treatment): *Trichostrongylus* (43%), *Teladorsagia* (17%), *Oesophagostomum*/*Chabertia* (27%) and *Haemonchus* (13%); whilst at D7, D14 and D21 (post-treatment), the following GIN genera were detected: *Trichostrongylus* (55%), *Teladorsagia* (7%), *Oesophagostomum*/*Chabertia* (26%) and *Haemonchus* (12%) on the group treated with natural anthelmintic.

In SF2, the following GIN genera were detected at D0 (pre-treatment): *Trichostrongylus* (47%), *Teladorsagia* (7%), *Oesophagostomum*/*Chabertia* (35%) and *Haemonchus* (11%); whilst at D7, D14 and D21 (post-treatment), the following GIN genera were detected: *Trichostrongylus* (46%), *Teladorsagia* (11%), *Oesophagostomum*/*Chabertia* (31%) and *Haemonchus* (12%) on the group treated with natural anthelmintic. On group treated with ivermectin very few numbers of *Trichostrongylus*, *Teladorsagia* and *Haemonchus* were found at D7, D14 and D21 (post-treatment).

### 3.2. Anthelmintic Efficacy

The results of the effectiveness of anthelmintic treatments related to natural anthelmintic administered at full and double dose and ivermectin drug administered at conventional dose, with the dosages used, the EPG (mean) of the groups and FECR (%) at different times (D) are shown in Table 1 and Table 2.

Following the WAAVP guidelines the ivermectin drug, used at conventional dosage, with FECR > 98 showed good anthelmintic efficacy while the natural vegetable mixture showed poor effectiveness. In fact, despite some decreases in the EPGs the FECR, at all two doses, was always maintained at values lower than those normally considered efficacy indices (highly effective FECR% > 98%, effective < 90–98%, moderately effective < 80–89%, poorly effective < 80%).

## 4. Discussion

The therapeutic potential possessed by medical plants makes phytotherapy an alternative to synthetic drugs. In recent years, research in the field of veterinary phytotherapies for the control of endoparasitosis in sheep has been strongly promoted [32,33]. Several published studies confirm the efficacy of some plants traditionally known for their anthelmintic properties in small ruminants, but most of the plants screened for anthelmintic activities are assessed through in vitro studies and only a few in vivo studies [33]. Moreover, the concentrations of potentially active substances in vitro do not always correspond to in vivo bioavailability.

The use of natural extracts should replace or complement control practices that rely solely on uninformed and repeated treatment of animals with drug in order to minimize dependence on synthetic drugs and to ensure and maintain high levels of animal health and welfare [32,34]. In addition to increasing incidence of parasite resistance against the available anthelmintics, the treatments with conventional anthelmintics has several drawbacks including the consumer concerns regarding drug residues in food products and in the environment, so it is important to identify a valid alternative based on predictive, preventive and systemic medical principles, which limit the sanitary and zootechnical risks under an acceptable threshold [34].

However, the present study reports that only ivermectin drug, at conventional dose, has proved to be effective against gastrointestinal infections in sheep, while the natural treatment was poorly effective, in vivo, without any apparent benefits to the animal health. Natural mixtures are rarely as effective as anthelmintic drug, but FECR of at least 40% formulation was would have been the desired result. However, the use these mixtures, combined with some control strategies (e.g., division and rotation of pastures, refugia strategies, supplementary feeding with bioactive forages, etc.), might gradually reduce the use of the drugs to an acceptable threshold. In agreement with Molento [32] it would be appropriate to identify new guidelines to evaluate the effectiveness of these new alternatives for parasite control.

## 5. Conclusions

This study confirmed that the scientific validation should be indispensable to ascertain the anthelmintic potential of plants. Therefore, it would be advisable to verify in vivo the efficacy of the complementary feeds registered for GIN control in sheep, in order to identify those with greater anthelmintic efficacy which could be used in farms in combination with control strategies.

In this way not only the health of the animals would be respected, but also the “feeling” of the final consumers, that consider animal welfare and respect for the environment essential principles of breeding, pay more attention to labels food and often choose carefully products from animals raised with natural systems, without the use of drugs.

## Figures and Tables

**Table 1:** Results of effectiveness of the anthelmintic treatments in sheep farm 1 (SF1) with animals treated with natural anthelmintic administered at 10 g/sheep as a single dose (dosages recommended by the manufacturer), eggs per gram (EPG, mean) and faecal egg count reduction (FECR, %) at different times (D).

Sheep Farm	Groups	D–7	D0	D7	D14		D21	
		EPG (mean)	EPG (mean)	EPG (mean)	EPG (mean)	FECR % (95% CI)	EPG (mean)	FECR (95% CI)
SF1	NG10 (10 g/sheep)	243	246	498	284	21.5 (16.2 to 26.2)	659	−33.9 (−20.4 to −38.5)
	CG1 (untreated)	242	240	362	362		492	

**Table 2:** Results of the effectiveness of the anthelmintic treatments in sheep farm 2 (SF2) with animals treated with natural anthelmintic administered at 20 g/sheep as a single dose (dosages recommended by the manufacturer under serious infestations) and animals treated with ivermectin drug administered as a single dose of 200 μg/kg/BW, EPG (mean) and FECR (%) at different times (D).

Sheep Farm	Groups	D–7	D0	D7	D14		D21	
		EPG (mean)	EPG (mean)	EPG (mean)	EPG (mean)	FECR % (95% CI)	EPG (mean)	FECR % (95% CI)
SF2	NG20 (20 g/sheep)	544	1023	2098	2194	−30.7 (−17.2 to −61.2)	1280	17.41 (11.0 to 51.0)
	CG2 (untreated)	543	944	1475	1678		1550	
	IVC (200 µg/kg)	544	1023	668	23	98.62 (96.8 to 99.5)	43	97.22 (93.4 to 99.1)
	CG2 (untreated)	543	944	1475	1678		1550	
